# Clinical Report on Cases Observed at the New Orleans Charity Hospital

**Published:** 1859-10

**Authors:** Austin Flint

**Affiliations:** Prof. of Clin. Med. in the New Orleans School of Medicine


### NEW YORK MONTHLY REVIEW,

AND

### BUFFALO MEDICAL JOURNAL.

VOL. 15. OCTOBER, 1859. NO. 5.

### ORIGINAL COMMUNICATIONS.

ART. I.—*Clinical Report on Cases observed at the New Orleans Charity Hospital.* By AUSTIN FLINT, M. D., Prof. of Clin. Med. in the New Orleans School of Medicine.

### MISCELLANEOUS CASES.

IN my three preceding reports I have taken up the cases of Pneumonia, of Diseases of the Heart, and of Typhoid Fever, observed at the Charity Hospital in 1858–9. One more report only can appear in the *New Orleans Medical News and Hospital Gazette* before another term of hospital service commences. This will, therefore, be my concluding report of the cases which came under observation during the last college-session. I shall select for this report several miscellaneous cases of interest, pursuing the same plan as heretofore; viz., giving a succinct account of the history of the cases, and appending to each case a few practical remarks.

The cases which I have selected for this report, are as follows:—1. A case of General Paralysis. 2. A case of Albuminuria. 3. A case of Acute Peritonitis. 4. A case of Ascites. 5. A case of Ascites. 6. A case of Acute Phthisis.

Case 1.—*General Paralysis, upper extremities especially affected. Phonation affected, without difficulty of respiration. Marked improvement.*

Adam Briber, German, aged 36, was an inmate of the hospital when my service commenced, Nov. 20th, 1858. He was admitted Oct. 12th.

The details of the previous history were not obtained, owing to his mental obtuseness, the labor of speaking, and his imperfect knowledge of our language. The paralytic affection had existed for two months prior to his admission, and he had suffered for two years from ailments which he called rheumatic.

The upper and lower extremities were incompletely paralyzed. He had kept the bed constantly since his admission. The loss of power over the upper extremities was greater than over the lower. The left upper extremity was more affected than the right. He was unable to raise the left arm, and was able to make only slight pressure with the hand. The lower limbs he moved freely in bed. The sense of tact and the impressibility to pain, on examination by palpation and with the æsthesiometer of Dr. Sieveking, appeared not to be impaired. The face was not paralyzed. The tongue was projected readily, and in a right line. The uvula did not incline to either side. There was almost complete aphonia. He spoke with difficulty in whispers, apparently from inability to command his breath sufficiently for this purpose. With a strong effort he could only say short words, such as *yes* or *no*, in a loud voice and in a grave tone. Breathing, however, was not labored, and the respiratory movements, on forced acts of respiration, of the chest and diaphragm, were marked. There was no difficulty in urination. The appetite and digestion were good. Aspect not morbid, but his countenance denoted imbecility. No cough. The pulse was normal in both wrists. The heart-sounds were normal. The spine was tender on pressure over the dorsal and lumbar portions; more over the latter than over the former; and the tenderness was more marked on the left than on the right side of the spinous processes. There was no projection at any point over the spine, nor curvature. The paralysis had been gradually developed, and could not be traced to any injury, nor to any exposure to lead.

Nov. 24th, the ward-nurse was directed to take this patient out of bed and cause him to sit up. The patient submitted to this with reluctance. He made no efforts to assist the nurse, but was as passive in his hands as a cadaver.

Dec. 3d. It was noted that the aspect of the patient was much improved. The plan of causing him to sit up daily had been persevered in, and he now sat up about four hours each day. The day previous, for the first time, he had walked across the ward, supported by the nurse. The nurse was directed to cause him to walk frequently.

Dec. 24th. The following is the note under this date,—"The improvement in this case is remarkable. He now sits up all the day; and he walks, with a little assistance. The countenance expresses much more intelligence. He is now desirous of being up as much as possible. He has acquired considerable power over the upper extremities. He speaks in a loud voice still, with difficulty, but he has improved in this respect. He was able yesterday to walk across the ward without assistance. The bowels are habitually constipated."

Jan. 8th, 1859. The improvement was still progressive. He was able to walk without difficulty. The improvement as regards the voice was marked.

Feb. 12th. The improvement had continued. A striking change had taken place in his aspect. His expression now denoted intelligence, brightness, and animation. The voice was almost completely recovered. He remained up from 5 A. M. to 6 P. M., and was able to make his own bed. The appetite and digestion were good, and the bowels were regular.

The hypo-phosphite of soda was prescribed soon after the case came under observation, in doses of a scruple three times daily. This remedy was continued for several weeks; and then discontinued, in order to observe whether the improvement was as marked without it as under its use. It was resumed after four weeks, my impression being that the progress was not as rapid after it was withdrawn as previously. This constituted the remedial treatment; excepting the administration of Huxham's tincture of bark in drachm doses, as a placebo, while the hypo-phosphite of soda was discontinued. Full diet was directed, and a portion of the time a little brandy.

*Remarks.*—The seat of the morbid condition of the nervous system giving rise to the paralysis in this case, was the spinal cord. This is inferred from the fact of the muscles of the four extremities being involved, and the exemption of the muscles supplied by the cranial nerves. The history of the case as regards the improvement, amounting nearly to recovery, while the patient was under observation, renders it probable that the morbid condition of the cord was merely functional, or, at least, that disorganizing lesions did not exist. This, however, it is to be considered, could not be predicated on the symptoms of the case; it is an inference from the result, and is, therefore, an after conclusion. The prognosis, in such instances, is to be based on the existence or non-existence of serious structural changes; and whether these exist, or not, cannot always be determined positively from the symptomatic phenomena. When there is room for doubt on this score, the practitioner is bound to act with the hope that the difficulty is merely functional, and consequently remediable. This secures for the patient the advantage of judicious measures of treatment, provided the hope be well founded, and exposes him to no damage if the hope turns out to be groundless. This practical precept is of immense importance; for functional paralysis if it continue, involves after a time, from its continuance, anatomical changes in the muscular and nervous fibres, precluding the hope of recovery, which at an earlier period might have been entertained. The practitioner should never lose sight of the fact that paralysis may become hopeless from its mere continuance. All ground of hope may be destroyed by the want of hope in season for measures of restoration to prove efficient. Paralytics not infrequently remain so, simply because the prognosis is unfavorable as judged by the law of probabilities. In other words, the want of success in a large proportion of cases discourages efforts, on the part of the physician and patient, which might have proved successful. If the paralysis be functional, recovery may be looked for with much confidence; and even when it depends on lesions of structure, a partial recovery is not always out of the question.

In the case now reported, the hypo-phosphite of soda was prescribed under the belief that phosphorous is a valuable remedy in certain affections of the nervous system. According to this remedy some influence, much more importance is to be attached to the moral and physical effects of the hygienic management. From non-exertion of the functions of body and mind, the patient had fallen into a state of apathy verging towards imbecility. This is the inevitable result of complete inactivity of sufficiently long duration. The patient had resigned himself to a hopeless condition, and he had become contented with a vegetative existence. He would never have taken the initiative in attempting to regain his lost powers. It was necessary to compel him to make the needed attempt. His mind was roused by taking him out of bed, and awakening his attention to himself and others. To develop his own interest in his recovery, was the first step, because his co-operation in the means of recovery was necessary. When this was accomplished, the means of recovery consisted mainly in the daily, persevering exercise of the paralyzed muscles. Let us consider, for a moment, the importance of this simple measure of treatment,—a measure undervalued, and thereby neglected, from its very simplicity.

We will assume that certain muscles have been paralyzed for some weeks or months, and the original morbid condition determining the paralysis, no longer exists; do the affected muscles, of their own accord, without any effort on the part of the patient, resume at once their former capability of action? That they do not, is exemplified by the effects of disuse of an upper or lower extremity in cases of fracture. After having been kept, from necessity, perfectly inactive for several weeks, the result is an incomplete paralysis; which is overcome, gradually, by exercise, until the powers which had been impaired simply by non-exertion, are fully restored. Without voluntary efforts on the part of the patient, this paralysis from disease would continue, and, at length, the capability of restoration would be lost. So, when paralysis is the result of a morbid condition of the nervous system: after this condition is removed, the affected muscles regain the ability to fulfill their functions only by means of exercise. If the morbid condition giving rise to the paralysis involve a lesion, for example an apoplectic extravasation, the immediate effect may be loss of power over certain muscles, which is more or less complete; but, after a time, by means of judiciously regulated and persevering exercise, considerable improvement may be effected, notwithstanding the cerebral lesion remains.

This principle is the basis of the successful treatment of paralysis. It is because this principle is not fully appreciated, and therefore not faithfully applied, that the treatment of paralysis is so generally unsuccessful. Comparatively speaking, remedial measures, such as electricity and strychnia, are of little value. Indeed, if these measures are relied upon to the exclusion of systematic, persistent efforts to regain the use of paralyzed muscles by means of exercise, they are more than valueless, since they stand in the way of the plan of treatment which offers the best chance for success. As this plan can only be effectually carried out with the co-operation of the patient, his interest is to be awakened, and his hopes encouraged, sufficiently to give the plan a fair trial.

The case now reported is highly interesting in another point of view. The faculty of phonation was nearly lost, while the muscles involved in respiration were but little, if at all, affected. The researches of Bernard have shown that the spinal accessory is to be regarded as the nerve of phonation. There was no evidence that the respiratory movements of the glottis were impaired in this case, but only the movements necessary for the production of the voice. As remarked by Bouillaud, some of the most valuable experiments to illustrate truths of physiology are instituted by disease; and in this instance, we have, as it were, an experimental demonstration of the discovery of Bernard.

CASE 2.—*Albuminuria—General Dropsy. Bright's Disease.*

Wm. Nixon, laborer, Irish, aged 22, admitted Nov. 17, 1858.

This patient stated that the commencement of his illness was three weeks before his admission. He had had excellent health, and had labored constantly for five years previously. He was treated in this hospital, in 1853, for bilious fever.

His present ailments commenced with the loss of appetite, occasional nausea, and debility. These symptoms progressively increased; but he kept about, although unable to labor, till the date of his admission, when he took to the bed. He improved after his admission, so that at the time the first note of the case was made, Nov. 20th, he was able to sit up and walk about.

He complained of no pain, but some uneasiness at the lower part of the chest in front on the right side, and in the lower limbs. The appetite had become tolerable. He had not had diarrhœa. He had noticed nothing unusual as regards the quantity or the appearance of the urine. A few days before his admission he had observed swelling of the lower extremities, and, shortly afterward, of the face. The legs were moderately œdematous. The upper extremities did not appear to be increased in size. The integument over the sternum pitted on pressure. There was considerable œdema of the face. The face was pallid, and the prolabia exsanguine. The pulse was 80, and feeble. The respirations were 20. There was deficiency of breath on exercise, but otherwise no dyspnœa. His vision was good.

On examination of the chest, the cardiac sounds were normal, and unattended by murmur. A small quantity of liquid was effused within the pleural sac on both sides, as determined by the variation of the limit of flatness in different positions of the body.

The urine was loaded with albumen, and presented a smoky appearance.

Nov. 21st. The day after the foregoing record was made diarrhœa occurred, no medicine having been prescribed. The dejections were frequent, large, and liquid. This diarrhœa continued for several days, and gradually diminished, no remedies being given to restrain it. He reported daily improvement in other respects. The œdema diminished. Epistaxis occurred repeatedly, but slight in degree. The diarrhœa at length ceased; and on the 29th of Nov. he felt sufficiently well to leave the hospital, and he was discharged.

The urine contained albumen in abundance at the time of his discharge, and the anœmic appearance continued.

The treatment consisted at first of the citrate of potassa, and afterward of the citrate of iron, with nutritious diet.

*Remarks.* This case presents no points of particular interest, but it will serve as a text for some practical remarks. Late researches have shown that Bright's disease, instead of having certain definite, uniform anatomical characters, involves various abnormal conditions of the kidney. In fact, it is evident that under this title are embraced several varieties of disease. Taking pathological anatomy as the stand-point, the diversity of morbid changes, their primary seat, their character and their effects, as investigated by Dr. George Johnson, and others, offer a most interesting and important field of study. These different conditions are to some extent represented, during life, by the various appearances presented by the casts derived from the renal tubes, and contained in the sedimentary deposits of the urine. I do not, however, propose to consider questions connected with the different forms of lesion which are found after death, but to offer some remarks on the cases generally included under the head of Bright's disease, as regarded in a clinical point of view.

A patient with general dropsy, and the urine highly or considerably albuminous, is generally said to be affected with Bright's disease. Now, these symptoms may disappear in the course of a few weeks, or even days, and not return; the patient's recovery being complete. This occurs when the affection is developed in pregnancy, and after scarlatina; but it also occurs when the affection is isolated from these connections, and apparently idiopathic. I have met with several instances of the latter description. If it be proper to apply the term Bright's disease to these cases, it is evident that the disease does not always involve permanent lesions of the kidney. An acute renal affection exists, in such instances, without leading to structural changes. The continuance of these symptoms for weeks and months, denotes a chronic affection which generally involves degeneration of structure in the kidney. These cases are sufficiently unpromising, but not necessarily so hopeless as they are sometimes regarded. I have met with cases in which great relief, and even apparent recovery, occurred under these circumstances. The practitioner should not give too discouraging a prognosis, nor consider all efforts in the way of management utterly hopeless. In the kidneys, as in other organs, nature has been bountiful in according a greater quantity of secreting tissue than is absolutely required; hence, these organs may be considerably damaged, and yet be able to fulfill their functions to the extent demanded for the preservation of life and tolerable health.

The evils and the dangers incident to the affection, so far as these are intelligible with our present knowledge, arise mainly from two sources, viz., the loss of albumen, in consequence of its elimination with the urine, and the retention in the blood of the urinary principles. Indications for treatment are derived from these two sources of pathological effects. Tonics, and a nutritious diet rich in albuminous constituents, are indicated by the former, and measures to prevent and relieve uræmia are indicated by the latter. Diarrhœa occurring spontaneously, as in the case reported, is to be regarded as an effort of nature to effect a vicarious excretion of urea through the intestinal canal. It is not, therefore, wise to attempt to arrest the discharges.

The events embraced in the clinical history of Bright's disease, and their pathological relations to the morbid condition of the kidney, would lead to a field of remark far too extended to be entered upon here. The case which has served as text for the foregoing general remarks, represents a class of cases often met with in hospital practice. Patients are admitted with anœmia, general dropsy, and debility ; and after remaining in hospital a short time, these symptoms are so far relieved that they feel able to return to labor, and desire to be discharged. The patients are usually careless as regards exposure, etc., and addicted to intemperance. They leave the hospital improved, but not cured ; nor is there much ground for the expectation of cure were they to remain ever so long. It would be, perhaps, as well for them to leave the hospital as to remain, if they were able or willing to pursue a proper hygienic course. But returning to their old ways of living, their ailments soon return ; and they are forced, after a time, again to seek refuge in a hospital. The time arrives when, if not carried off by pericarditis, or some other serous inflammation, or uræmic coma, the dropsy becomes permanent; the powers of life are too much exhausted to admit of even temporary improvement, and the disease proves fatal by slow asthenia.

### Case 3.—*Acute Peritonitis. Recovery.*

John Newman, aged 21, was admitted Nov. 21st, with intermittent fever. The sulphate of quinia was prescribed, and he reported daily improvement, being always up and dressed, and going out of doors. During the night of Dec. 1st, he was attacked with severe pain in the abdomen. At my morning visit I found him suffering greatly from pain, which he referred to the upper part of the abdomen. The abdomen was exceedingly tender on pressure, and the suffering from pressure proportionate to the force employed. There was rigidity of the recti muscles, but no enlargement of abdomen. The pulse was frequent, and the countenance expressed anxiety and suffering. A grain of the sulphate of morphine was at once given, with directions to repeat this remedy in half-grain doses every four or six hours, if the pain continued. During the day and night three grains of the sulphate of morphia were given. This quantity sufficed to render him comparatively comfortable. During the day and night he vomited repeatedly, the acts of vomiting causing excruciating pain. He had also several dejections from the bowels.

Dec. 3d. The pain continued, but was less severe, and he was moderately under the influence of the remedy. The pain was lancinating in character. Tenderness was marked over the entire abdomen, but without tympanitic distention, or marked rigidity. Coughing or moving occasioned great pain. The pulse was 128, small, vibratory. The skin was hot and dry. The decubitus was on the back, with the knees drawn up much of the time, but not constantly. The respirations were 30, and the respiratory movements costal. The urine was not albuminous. The treatment directed was, the sulphate of morphia gr. ss. every three hours.

Dec. 4. He reported better. From a misapprehension as regards the treatment directed, he got only half a grain of the sulphate of morphia. Decubitus on the side, with the knees drawn up. Tenderness over the abdomen continued, but without tympanitic distension. Muscular rigidity of the muscular walls was observed when pressure was made. The pulse was 100; the respirations 28, and costal. No dejection since the night previous, but vomiting had occurred several times. Auscultation of the abdomen did not disclose friction-sounds, but borborygmi in abundance. On being questioned, the patient stated that he had frequent erections, with strong desire for coitus. These erotic paroxysms continued for about five minutes. The treatment directed was, half a grain of the sulphate of morphia every three hours.

Dec. 5th. The patient reported free from pain. The abdomen was moderately tympanitic, and tender on pressure. No dejection. Priapism had not occurred. The pulse was 76, and the respirations 16. Half a grain of the sulphate of morphia was directed every four or six hours.

Dec. 6th. The patient was comfortable. Moderate tympanites continued, with tenderness on firm pressure. The pulse was 70, and the respirations 20. No priapism. Half a grain of the sulphate of morphia was directed every six hours. Essence of beef and milk for diet.

Dec. 7th. The patient complained of pain in the abdomen. The abdominal tenderness was increased. The pulse was 66, and the respirations 16. No dejection. Half a grain of morphia was directed every four hours. Diet as before.

Dec. 8th. The patient was free from pain. No dejection. The pulse was 64, and the respirations 16. The treatment was continued.

Dec. 10th. The patient reported daily better. No dejection since the 4th inst. Pulse 78, and respirations 20. The treatment was continued.

Dec. 14th. The patient complained of no pain, except with movements of the body. Tenderness on deep pressure continued, with some rigidity. No dejection had occurred since the 4th inst. (ten days), and he was anxious to have a cathartic medicine prescribed. For several days he had been desirous of sitting up, and was permitted to do so on this date. The sulphate of morphia was reduced to a quarter of a grain three times daily.

Dec. 15th. A solid dejection had occurred, the first since the 4th inst. The patient was able to sit up for a short time. Treatment was continued.

Dec. 24th. Up to this date the patient had continued to convalesce. The sulphate of morphia had been continued. Milk-punch had been given, and improved diet allowed. During the night preceding the present date he was seized with diarrhœa, which was relieved by an enema of laudanum. The pulse was 60, and the respirations 16.

Dec. 29th. Diarrhœa recurred, but was promptly relieved by an enema of laudanum. Five grains of the sulphate of quinia and two grains of opium were directed three times daily, with milk-punch and nutritious diet.

Jan. 8th, 1859. The patient had continued to convalesce, excepting occasional slight recurrences of diarrhœa. The opium was diminished on this date to one grain three times daily, and in a few days withdrawn.

The patient was discharged quite well, January 12th.

*Remarks.* This case is of interest on account of the infrequency of acute, idiopathic peritonitis in the male sex. If we exclude the cases in which the disease is traumatic, and those in which it is due to perforation of the intestine, peritonitis is certainly one of the rarest of diseases. It is less infrequent in females than in males, exclusive of the cases in which it is developed in connection with the puerperal state. In this case the disease was developed in the hospital, without any appreciable cause. It was apparently purely idiopathic.

The case is interesting, as illustrating the opiate plan of treatment. The sulphate of morphia, opium, and laudanum by enema, were the only remedies prescribed, excepting the sulphate of quinia after convalescence was established. The treatment, thus, was exclusively opiate. Mercury, blood-letting, and counter-irritation were not employed. Cathartics, or laxatives, were not given, even after convalescence; and the bowels were allowed to remain unmoved for eleven days. The quantity of opiate remedies given, however, was not excessive, three grains of the sulphate of morphia on the first day being the maximum; and afterward, during the career of the disease, about two grains daily, constituting the treatment. So far as the issue and progress of the case furnish evidence of successful treatment, it was certainly all that could be expected. The acute pain ceased when the patient was brought under the influence of the anodyne; the tympanitic distension of the abdomen was not great; the pulse, which on the second day was 128, fell on the fourth day to 100, and on the fifth day to 76. As is not uncommon in this disease, after the intensity of the inflammation had ceased the pulse fell below the average frequency; on the eighth day it was 64. I have observed the pulse under these circumstances to become irregular, as well as infrequent. The patient was able to sit up on the fifteenth day. Convalescence was only interrupted by occasional attacks of diarrhœa, which were promptly relieved by enemas of laudanum. The convalescence, however, as is usual, was slow; and the patient was not well enough to be discharged until forty-three days after the date of the attack.

The method of treating acute peritonitis exclusively by opium, was inaugurated (to use the term just now in vogue) by Prof. Alonzo Clark. Prof. Clark demonstrated, some ten years ago, the power of this remedy in controlling the disease, and the remarkable tolerance of the remedy in certain cases. Observation by himself and others since the publication of the reports of cases of puerperal peritonitis treated at the Bellevue Hospital, has shown that the large doses of opium given in these cases are by no means always required. The plan of treatment is to induce a narcotic effect sufficiently to relieve entirely the abdominal pain, and to maintain this effect steadily throughout the career of the disease. The difference in the susceptibility to this effect is so great in different cases, that it is out of the question to formularize the administration of the opiate remedy. Small doses will suffice in some cases, and in other cases enormous quantities are requisite. The doses and their repetitions are to be determined in individual cases by the effect, the object being to carry the remedy to the extent of subduing pain; but, of course, taking care to avoid a dangerous degree of narcotism. The form of opiate is also to be determined by circumstances in individual cases. If opium be used, a liquid form instead of the solid gum is to be preferred, because the doses are more easily regulated by the effects. The salts of morphia are probably not less efficacious, and they are administered more easily than other forms. These may be placed dry upon the tongue, if, as is sometimes the case, irritability of the stomach conflicts with the administration of remedies by the mouth. They may also be employed efficiently by sprinkling upon a blistered surface denuded of the cuticle.

Prof. Clark has rendered a great service to practical medicine and to humanity by establishing the merits of this method of treating acute peritonitis. If pursued judiciously and boldly, a large proportion of the cases which, judged by former experience, would have otherwise ended fatally, are brought to a favorable termination. The greater success in the management, however, it must be confessed, may be in part owing to the discontinuance of measures which were injurious. In this light we must regard blood-letting and cathartics. As regards blood-letting, a fair and ready way of placing before the mind its theoretical applicability to the treatment of peritonitis, is to consider the extent of surface inflamed in this disease, and the loss of blood-constituents involved in the exuded products of inflammation. The condition of a patient attacked with peritonitis, is not unlike that of a person after a scald, or burn, extending over a large portion of the external surface of the body. The symptoms are analogous in the two cases, and death in both occurs by asthenia. Blood-letting is as appropriate in the one case as in the other. Of cathartics, it is only necessary to say that they conflict with the first and great indication in the treatment of all inflammations, viz., to maintain, as far as possible, repose of the parts inflamed. The value of opiates in cases of peritonitis consist, in fact, of the arrest of the peristaltic movements of the intestines. These remedies have held so prominent a place in therapeutics for the last half-century, that it requires some moral courage on the part of the practitioner to permit the bowels to remain constipated for a fortnight or longer, and to resist the importunities of patient and friends for opening medicine.

It is stated in the notes of this case, that the patient, during the progress of the disease, experienced frequent erections, and a strong desire for coitus. This belongs to the history of acute peritonitis in certain cases, how frequently is to be yet ascertained. In a case which came under the observation of my friend and former colleague, Prof. Rogers, of Louisville, the patient consummated the act of coitus but a short time before his death. This fact was communicated to Prof. R. by friends of the patient, in order to protect the reputation of the widow should it so happen that pregnancy followed the connection. Whether this be a common or only an accidental effect of the disease, I am not prepared to say.

Case 4.—*Ascites, Cirrhosis. The liquid rapidly absorbed, while the patient was taking only the Huxham's tincture of bark. Advantage of the expectant system in certain cases illustrated, and the post-hoc-propter-hoc method of reasoning.*

The above caption is transferred from the hospital record-book, where it was placed without any idea at the time of reporting the case. It expresses the several points of view in which the case is of interest.

Elie Monet, Frenchman, carpenter, aged 36, was admitted Dec. 28th, 1858. He stated that he had been for several years addicted to intemperance, but had not drunk freely for the preceding month and a half. He had been in ill health for six months. For over four months he had frequent attacks of intermittent fever; these had not recurred for the last month and a half. He noticed swelling of the abdomen and, about the same time, of the feet, a month before his admission.

Dec. 29th. The abdomen was greatly distended, and fluctuation sufficiently distinct. The feet and lower limbs were moderately œdematous. There was no œdema of the face. The body was emaciated. He measured ver the abdomen, just above the umbilicus, forty-one inches. There was no febrile movement. The urine was not albuminous. The bowels were open, but no diarrhœa.

A quarter of a grain of elaterium was directed every four hours, with full diet, but restriction as regards liquids.

Dec. 30. The patient had had twenty liquid dejections, and had vomited twice. On measurement, the circumference of the abdomen was reduced an inch. The pulse was 120, and the respirations 30.

Full diet, milk-punch, and no medicine, were directed.

Dec. 31. Reported more comfortable.

A quarter of a grain of elaterium every four hours was directed.

Jan. 1, 1859. Reported comfortable. He had had eight copious liquid dejections. Abdomen diminished an inch, measuring now thirty-nine inches.

Huxham's tincture of bark, ʒ i. three times daily, was prescribed, and a dry diet.

Jan. 2. Reported the same. A quarter of a grain of elaterium three times was prescribed.

Jan. 3. The patent had had ten liquid dejections. Nausea and vomiting had followed each dose of the medicine. The abdomen measured half an inch more than on the 1st inst., viz., 39½ inches.

The urine of the patient presented purpurine in abundance.

Full diet was directed, and no medicine.

Jan. 20. No records were made after Jan. 3d up to this date, when the following was noted: "The elaterium was not repeated after the date of the last record, in consequence of the prostration which it induced, and the non-diminution of the size of the abdomen after the last doses of this remedy on the 2d inst. My intention was to discontinue medicine for a few days, and then resort to tapping. As a placebo, in the meantime, the Huxham's tincture, in doses of a drachm three times daily, was prescribed. The patient has steadily improved. He reports daily better, and his aspect denotes marked improvement. The abdomen has daily diminished in size. Under these circumstances, the Huxham's tincture is continued, with full diet. He is under the impression that the medicine which he takes is doing wonders for him!

"On examination of the abdomen, the enlargement is mainly at the lower part; it bags down; and the navel is quite low, falling below the level of the crest of the ilium. Measured just above the pelvis, the circumference is 31½ inches."

Jan. 28. The patient had continued to improve. The abdomen was progressively diminishing in size. The Huxham's tincture and full diet continued to constitute the treatment.

Jan. 30. The patient declared that he was able to go to work, and was therefore discharged. The abdomen was pendulous, and no liquid was discoverable, either by palpation or percussion. He measured over the navel, 30 inches. The liver was evidently below the usual size, as determined by palpation and percussion. The patient, who imputed the cure to the medicine, was supplied, at his request, with a quantity, in order to continue its use after his discharge.

*Remarks.* The points of interest in this case, as already stated, are embraced in the caption. The elaterium, which at first diminished the ascites, at length ceased to have this effect, and could not be borne. Tapping was resolved upon, and postponed merely for convenience, the occasion not being urgent. The improvement during the delay caused the operation to be abandoned; and inasmuch as this improvement was daily going on, when the Huxham's tincture was given as a placebo, no other remedy was prescribed. The patient was discharged sufficiently well to return to work, after having been in hospital thirty-two days.

The patient was supposed to be affected with cirrhosis of the liver. The habits of the patient, the history of the case, and the contraction of the liver as ascertained after the ascites had disappeared, rendered this diagnosis almost positive. Nor does the progress of the case disprove the correctness of the diagnosis. I have met with another instance in which, in like manner, the ascites disappeared, and the patient was exempt from it for several weeks; but it afterward returned, and the disease proved fatal. The explanation of the improvement and apparent recovery, is as follows: The cirrhosed condition of the liver was not intrinsically enough to occasion dropsy under favorable hygienic circumstances. Poor living, dissipation, and the effects of intermittent fever, co-operated with the hepatic lesion in producing the ascites. These accessory causes being removed, the dropsy temporarily disappeared, notwithstanding the cirrhosis. On returning to labor, however, and resuming his old habits of life, the cirrhosis would be likely to progress; and probably, ere this time, the ascites has returned, and the disease has, perhaps, ended fatally.

Case 5.—*Ascites, Cirrhosis. Fatal. Autopsy.*

In the following case, the same affections as in the preceding case led to a different termination. This case may be considered as furnishing the complement of the after-history in the preceding case.

Francis Picard, Frenchman, laborer, aged 35, was admitted February 3d, 1859.

The abdomen was enormously distended. In the recumbent position on the back, the whole abdomen was resonant on percussion. In the sitting posture, flatness on percussion existed at the lower part, extending upward several inches above the pubis. The level of the flatness varied with the position of the body, showing the presence of liquid. Much of the enormous distention, however, appeared to be from tympanites. He was pallid, emaciated, and extremely feeble. The previous history was not ascertained. He stated that he had been under medical treatment, and had been recently salivated. He had little appetite, but much thirst. The urine was abundant, and not albuminous. There was no œdema of the lower extremities. The pulse was not accelerated, but very feeble. Physical exploration furnished no evidence of cardiac disease. The patient failed rapidly, and died on the sixth day after his admission.

On examination after death, the peritoneal sac contained a large bucketful of transparent, yellowish serum. There was no exuded lymph, and the peritoneal membrane was not opaque. The stomach and small intestines were greatly distended with gas. The liver was diminished in size, and its edges blunted. It was hard and resisting, and portions presented a yellowish granulated appearance, the different order of vessels not being distinguishable. The surface was smooth, not nodulated.

The kidneys presented externally and on section a normal appearance. The heart was normal.

*Remarks.* The occurrence of ascites in cirrhosis is due to the mechanical obstruction to the passage of blood in the liver from the portal to the hepatic system of veins. Congestion of the portal veins follows, and venous transudation into the peritoneal sac. Clinical observation shows that the occurrence of ascites and its degree are not always proportionate to the amount of hepatic disease. The dropsy may be considerable, or great, with a moderate amount of cirrhosis; and, on the other hand, it is not always great when the liver is much affected. The explanation of this want of correspondence between the lesion and its effects, involves various accessory circumstances; one of which is the relief afforded to the portal circulation by means of anastamosis with the systemic venous system. On this subject, M. Sappey has lately contributed a memoir to the French Academy of Medicine, which is of interest in this connection. The following are the conclusions of M. Sappey, quoted from the *Archives de Médicine*, Avril, 1859.

1. There is no evidence of the persistance of the umbilical vein in the adult; and all the facts which have been considered as proving such persistance, must be regarded as examples of dilatation and hypertrophy of one of the small veins contained in the suspensory ligament of the liver.

2. This small vein, in becoming dilated and hypertrophied, leads to the dilatation and hypertrophy of the veins with which it anastamoses, and thus is the point of departure of a large collateral channel, extending from the sinus of the portal vein to the principal vein of the lower extremity.

3. In this collateral channel the blood flows from above downward, and not from below upward as all authors now and hitherto have taught.

4. This channel embraces sometimes the sub-aponeurotic and sometimes the sub-cutaneous veins of the abdomen.

5. When it embraces the sub-aponeurotic veins, neither varices nor varicose tumors are developed in their tract; but when it embraces the sub-cutaneous veins, one or more of these tumors is always produced.

6. The venous current flowing from the liver toward the crural vein occasions a thrill (*frémissement*), perceptible to the hand, and a continuous murmur perceived through the stethoscope.

7. Finally, the existence of this current must be considered, in the great majority of cases, as a favorable symptom, since it lessens the liability to ascites, although it points to confirmed and incurable cirrhosis.

CASE 6.—*Acute Phthisis. Collapse or Atelectasis of the lower lobe of left lung. Fatal. Autopsy.*

Franz Ziegler, aged 58, shoemaker, was admitted January 3d, 1859.

He stated that he had had habitual cough for nine years. For the last five years he had felt want of breath on exercise. He had been repeatedly confined to the bed for several days, after, apparently, contracting a cold.

On his admission, he was obliged to keep the bed; the cough was frequent and severe, with considerable muco-purulent expectoration; and the suffering from want of breath was a prominent symptom. The face was pallid and tumefied. There was moderate œdema of the lower extremities. The appetite was tolerable. Pulse, 120.

*Physical signs:* The summit of the left side was depressed. The respiration was abdominal and inferior costal. The respirations were labored, and varied from 40 to 60 per minute. Marked relative dullness on percussion, existed over the left scapula and the upper third of the left side in front. In the latter situation the dullness approached to flatness. Dry râles, sonorous and sibilant, were diffused everywhere over the chest; but this did not present a well-marked bronchial respiration from being apparent over the left scapula. At the summit of the left side in front, the respiratory murmur was either suppressed, or drowned in the râles. Well-marked bronchophony and the bronchial whisper existed over the left scapula. The sounds of the heart were pure.

The iodide of potassium, grs. x. twice daily, was prescribed, with the syrup of morphia, and nutritious diet.

Jan. 13th. The patient died suddenly. He had failed for several days, and on this date he was extremely feeble. In the afternoon he got up to go to the water-closet, fell on the floor, and died almost instantly.

On examination after death, old and very firm pleuritic adhesions existed on both sides. In place of the lower lobe of the left lung, there existed a small triangular mass about two inches in length, and the same in width at the base. On section, it presented the appearance of carnified lung. It did not occur to me to attempt to inflate it before cutting into it. There was no deposit of tubercle in this collapsed lobe. The upper lobe of the lung was contracted and solidified by disseminated miliary tubercles. Slight crepitation was produced by pressure on the lower part of this lobe. The bronchial tubes were moderately dilated. No aggregation of the tubercles into masses, nor softening, nor excavations. The left bronchus was dilated, exceeding the right in caliber by about as much as the right bronchus normally exceeds the left. The volume of the right lung appeared to have been increased, but no enlargement of the cells apparent to the naked eye. The upper lobe of this lung contained numerous miliary tubercles, but not in sufficient abundance to produce palpable solidification. The lower lobe contained a few tubercles,—not seen, but felt when the finger was passed over the incised surfaces. The heart was moderately enlarged, but no valvular lesions. The aorta above the valves was somewhat dilated, and roughened with atheromatous deposit.

*Remarks.* This case furnishes an illustration of acute phthisis, which is characterized by the deposit of miliary tubercles, or semi-transparent granulations, in great abundance, destroying life by their accumulation, without being aggregated into masses, softening, and ending in excavations. These processes of ordinary tuberculosis sometimes take place with great rapidity, giving rise to rapid or galloping consumption, in distinction from acute phthisis. The latter is distinguished by the non-occurrence of tuberculous masses and cavities. It is not easy to determine, from the previous history, when the deposit of tubercles commenced; but that it had been going on for the nine years during which cough had existed, is by no means probable. It was probably a recent event, having been preceded by chronic bronchitis of many years' duration.

The absence of the lower lobe of the left lung, except in a rudimentary state, is a curious feature of the case. Had this lobe never expanded; or had collapse occurred as a result of bronchial obstruction? The latter is, perhaps, the more probable supposition. The want of this lobe would account for the deficiency of breath on exercise, which the patient had experienced for a long time.

